# ATP-Binding Cassette Transporter of Clinical Significance: Sideroblastic Anemia

**DOI:** 10.3390/jpm14060636

**Published:** 2024-06-14

**Authors:** John O. Ogunbileje, Neil Harris, Tung Wynn, Reema Kashif, Brian Stover, Bremansu Osa-Andrews

**Affiliations:** 1 School of Medicine, Indiana University, Indianapolis, IN 46202, USA; jooyelak@iu.edu; 2 College of Medicine, University of Florida, Gainesville, FL 32611, USA

**Keywords:** anemia, sideroblastic anemia, ATP-binding cassette transporter, mutation, ringed sideroblasts, mitochondria

## Abstract

The ATP-binding cassette (ABC) transporters are a vast group of 48 membrane proteins, some of which are of notable physiological and clinical importance. Some ABC transporters are involved in functions such as the transport of chloride ions, bilirubin, reproductive hormones, cholesterol, and iron. Consequently, genetic or physiological disruption in these functions is manifested in various disease processes like cystic fibrosis, Tangier disease, and sideroblastic anemia. Among other etiologies, primary sideroblastic anemia results from a genetic mutation in the ATP-binding cassette-7 (ABCB7), a member of the ABC transporter family. There are not many articles specifically tackling the disease processes caused by ABC transporters in detail. Some testing methodologies previously reported in the available literature for investigating sideroblastic anemia need updating. Here, we expound on the relevance of ABCB7 as a clinically important ABC transporter and a rare participant in the disease process of Sideroblastic anemia. The other genetic and secondary etiologies of sideroblastic anemia, which do not involve mutations in the ABCB7 protein, are also described. We review the pathophysiology, clinical course, symptoms, diagnosis, and treatment of sideroblastic anemia with a focus on modern technologies for laboratory testing.

## 1. Introduction

Sideroblastic anemia is a rare hematological disease caused by impairment in heme synthesis. Molecular disarrangement of the ABCB7 transporter (part of a large superfamily of ATP-dependent transmembrane proteins) [1] and genetic mutations in other proteins, as well as environmental factors, have been widely established as etiologies of the disease. Expounding on updates in diagnostic strategies, work-up algorithms, monitoring, and treatment options is imperative as medical science gravitates toward specialized and personalized medicine. Research scientists will be able to link well-described proteins like ABCB7 transporters with their clinical significance and involvement in the disease processes. Laboratorians and clinicians will be exposed to testing tools for diagnosing and monitoring sideroblastic anemia. The incorporation of a clinical case offers a vivid appreciation of best practices for the interpretation of tests with respect to the management of a typical sideroblastic anemia patient. The clinical case unwinds the diagnostic criteria, appropriate testing, treatment, and monitoring of the disease.

## 2. General Background and Etiology of Sideroblastic Anemia

**Figure 1:**
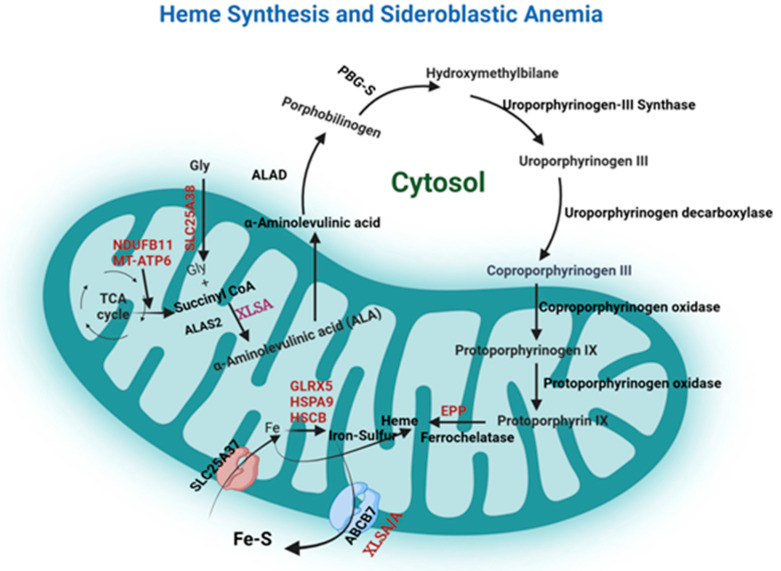
Heme biosynthetic pathway in erythroid cells and the role of ATP-binding cassette transporter B7 (ABCB7 gene): The first enzyme of heme synthesis is 5-aminolevulinate synthase (ALAS2), which is the rate-limiting enzyme. In the presence of pyridoxal phosphate, it condenses glycine and succinyl-coenzyme to form ALA. Mutations in the erythroid-specific ALAS2 gene cause X-linked sideroblastic anemia (XLSA) by impairing the synthesis of delta-aminolevulinic acid (ALA). ALA is transported out of the mitochondria into the cytosol to continue the heme synthesis. Ultimately, heme synthesis involves the incorporation of ferrous iron into protoporphyrinogen. However, hereditary sideroblastic anemia can affect genes that encode proteins involved in the synthesis, trafficking, or incorporation of iron–sulfur clusters, such as ALAS2, HSPA9, SLC25A38, GLRX5, or ABCB7. In particular, ABCB7 is crucial for hematopoiesis and is located at Xp13.3. The ABCB7 gene encodes a mitochondrial transporter of the Fe–S cluster. These clusters are initially synthesized as Fe-S intermediates within the mitochondria and subsequently transported to the cytoplasm for maturation15 by the mitochondrial transporter ABCB7. ABCB7 mutations cause a rare recessive X-linked sideroblastic anemia with ataxia (XLSA/A) disorder.

Sideroblastic anemia is a group of bone marrow disorders characterized by the pathological accumulation of iron in the mitochondria of erythroblasts due to impaired heme synthesis [2]. The pathogenesis stems from the inability to incorporate iron into protoporphyrin during heme synthesis (Figure 1, Table 1), resulting in excess accumulation of iron in the mitochondria, subsequently reducing erythropoiesis and causing anemia [3]. The increased number of ringed sideroblasts in the bone marrow exacerbates the suppression of hematopoiesis [3]. Sideroblastic anemia can be categorized into primary (congenital) and secondary (acquired) disorders [2,4,5]. Each of these has distinct etiological factors and mechanisms that might sometimes be heterogeneous [4].

## 3. Primary (Congenital) Sideroblastic Anemia

Primary sideroblastic anemias are inherited mitochondrial dysfunctions caused by impaired heme biosynthesis pathway, iron–sulfur cluster biogenesis, and generalized mitochondrial protein synthesis [2,4,6]. The most common primary sideroblastic anemia cases demonstrate an X-linked recessive inheritance (XLSA) pattern [2,4]. Unbalanced lyonization could explain why the recessive variant, which inadvertently affects only women in an X-linked inheritance pattern, is the most prominently seen. Inactivation of one X-chromosome could predispose females in a family to carrying only the mutated gene but also ensure females are not twice as affected as males [7]. XLSA is caused by mutations in the erythroid-specific ALAS2 gene (Figure 1), which codes for delta-aminolevulinic acid (ALA) synthase [2,4]. ALAS2, the rate-limiting enzyme (Figure 1), is the first enzyme of heme synthesis. It condenses glycine and succinyl-coenzyme A to form ALA in the presence of pyridoxal phosphate [2,8]. The deficiency of this enzyme impaired normal erythropoiesis (Figure 1), resulting in iron accumulation within mitochondria and ringed sideroblast formation [5]. There is also a separate autosomal recessive sideroblastic anemia caused by mutations in SLC25A38, a glycine amino acid carrier responsible for glycine import into mitochondria.

Other forms of inherited sideroblastic anemia are caused by mutations in genes responsible for iron–sulfur cluster (ISC) biogenesis and generalized mitochondrial protein synthesis [2]. However, they are less common compared with XLSA [2]. Iron–sulfur clusters are critical cofactors needed for various cellular processes and mitochondrial metabolism, including energy production, DNA repair, and the regulation of gene expression [2,3,4]. Hereditary sideroblastic anemia can affect genes that encode proteins involved in the synthesis, trafficking, or incorporation of iron–sulfur clusters, such as ALAS2, HSPA9, SLC25A38, GLRX5, or ABCB7 (Figure 1) [4,6]. Mutations of HSPA9 are the most common mitochondrial iron–sulfur cluster (ISC) biogenesis [2,9]. HSPA9 and HSCB transfer nascent 2Fe-2S ISC to mitochondrial GLRX5 for further distribution [2]. Mutations of these genes lead to nonsyndromic microcytic to normocytic sideroblastic anemia [2]. Furthermore, adenosine triphosphate ATP-binding cassette B7 (ABCB7) has been identified to be critical in the pathogenesis of sideroblastic anemia [4,10]. Several reports have shown that the mutations in the ABCB7 gene are seen in individuals with X-linked sideroblastic anemia with ataxia (XLSA/A) (Figure 1), highlighting its importance in erythropoiesis [2,10]. Interestingly, even partial loss-of-function mutations in ABCB7 cause a rare syndromic form of microcytic X-linked CSA associated with cerebellar ataxia (XLSA/A) [2].

## 4. Acquired (Secondary) Sideroblastic Anemia

**Figure 2:**
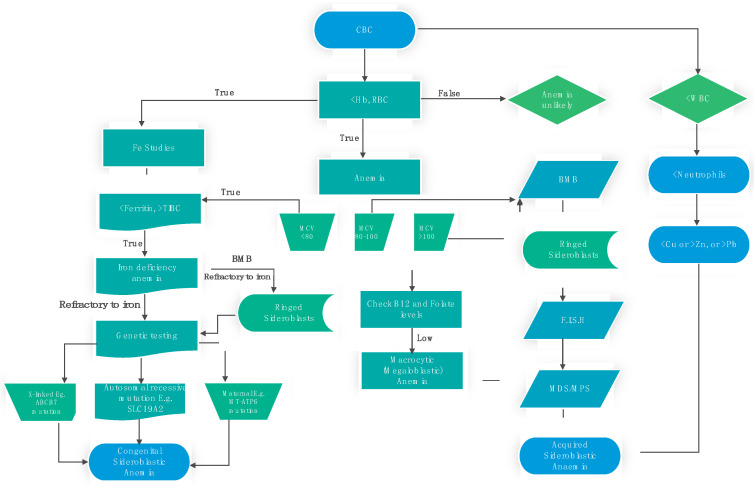
Simplified schematic for the diagnostic workup of sideroblastic anemia. The initial laboratory test performed to investigate suspected anemia is the CBC, in which normal to high Hb and RBC rule out the possibility of anemia. If Hb, RBC, and MCV are low, iron studies are recommended, and iron deficiency anemia should be treated if indicated. In the event of refractory iron deficiency anemia, the presence of ring sideroblasts on BMB and positive genetic testing are diagnostic of congenital sideroblastic anemia. Normal or higher MCV should also lead to BMB, and the presence of ring sideroblasts indicates acquired sideroblastic anemia. FISH is useful to determine if sideroblastic anemia is secondary to MDS. On the other hand, leukopenia and neutropenia in the setting of low copper, high zinc, or high lead also indicate secondary sideroblastic anemia. CBC, complete blood count; BMB, bone marrow biopsy; MCV, mean corpuscular volume, B12, vitamin B12; Hb, hemoglobin; FISH, Fluorescence In Situ Hybridization; MDS, myelodysplastic syndrome; MPS, myeloproliferative syndrome; WBC, white blood cells; RBC, red blood cells.

Acquired sideroblastic anemia is grouped into those that are reversible, such as those from environmental factors, and those that are due to clonal hematopoiesis, such as in subtypes of myeloproliferative neoplasm (MPN), myelodysplastic syndromes (MDS). and acute myeloid leukemia [3,4,11]. Acquired sideroblastic anemia can result from exposure to toxic metals (lead and zinc overdose), certain medications (isoniazid, pyrazinamide, and chloramphenicol), nutritional deficiencies (copper deficiency and vitamin B6 deficiency), and alcohol abuse (Table 1, Figure 2) [3,4]. The mechanism of trace metal-induced sideroblastic anemia is multifactorial but closely linked together. ALAS2, a prominent enzyme in heme biosynthesis, depends on zinc as a cofactor for its activity. In lead poisoning, lead competes with zinc for the active site of ALAS2 [12]. Concomitantly, delta aminolaevulinic acid accumulates, and heme synthesis is truncated. A second biochemical route for lead poisoning-induced sideroblastic anemia is through the enzyme ferrochelatase, the final enzyme in the heme synthesis pathway, which incorporates ferrous iron into the heme molecule. Porphyrins increase, but ring sideroblasts are typically not associated with lead poisoning. Two copper-requiring proteins are critical in the metabolism and transport of iron. Ceruloplasmin is involved in the conversion of ferrous to ferric iron required for the transport of transferrin. Conversely, cytochrome oxidase is required for the reversal of ferric back to ferrous iron in the tissues, which enables iron to be incorporated into heme [13]. Hypocupremia, therefore, plays a key role in impairing heme synthesis and ultimately in causing sideroblastic anemia. The presence of iron that is not incorporable into heme leads to the formation of ringed sideroblasts in erythrocytes. The clinical features of hypocupremia resemble those of myelodysplastic disorders in that it induces dysplasia. WBC differentials often reveal neutropenia, and increased hematogones are visible in flow cytometry. Copper supplementation often resolves ring sideroblasts but not necessarily the neurological symptoms [4]. Key culprits in the development of hypocupremia are low ingestion in diet and the malabsorption of copper. Zinc supplements are commonly prescribed for the management of patients with Wilson’s disease, a genetic condition that results in excessive levels of unbound copper. This zinc treatment is not only possible due to the displacement of copper by the more reactive and electropositive zinc, but also zinc stimulates the release of metallothionine, which hinders the intestinal absorption of copper while increasing fecal elimination of copper [4,14]. Consequently, zinc overdose frequently causes copper deficiency. A deficiency in pyridoxal phosphate, a cofactor of ALAS2, impairs the enzyme and diminishes heme biosynthesis [3,4], resulting in hypochromic and microcytic anemia in addition to the formation of ring sideroblasts. Hypothermia has also been reported to interfere with mitochondria metabolism, resulting in sideroblastic anemia [3]. Peripheral blood smears commonly reveal ring sideroblasts in the blood of about 30% of chronic alcohol consumers [4]. Ring sideroblasts, associated with alcohol-induced sideroblastic anemia, may disappear following the cessation of alcohol consumption. MCV levels in the setting of alcohol abuse typically range from normal to high. Another common observation on peripheral blood smears is double populations of RBCs. In addition to ring sideroblasts, RBC vacuolization is observed in bone marrow biopsy in alcohol abuse [4,15]. Alcohol interferes with pyridoxine metabolism by directly and toxically impairing the activity of delta-aminolaevulinic acid synthase, a key enzyme involved in heme synthesis [4]. Generally, acquired sideroblastic anemia can be inverted after the removal of the alcoholic stimulant but may linger in the setting of diminished folate stores [4].

Clonal sideroblastic anemias are associated with ring sideroblasts with perinuclear mitochondrial iron accumulation [4,11]. Specifically, they are seen in some subtypes of MDS, MPN, MDS/MPN overlap syndromes, and acute myeloid leukemia (AML). According to the World Health Organization (WHO) 2017 classification, the presence of ring sideroblasts in bone marrow (BM) is a diagnostic criterion for MDS with ring sideroblasts (MDS-RS) and MDS/MPN with RS and thrombocytosis (MDS/MPN-RS-T) [4,11,16]. Mutations in SF3B1 are seen in ≥80% of patients with MDS-RS [4,11]. Though MDS-RS is the most commonly acquired sideroblastic anemia, it is a lower-risk MDS [4,11].

## 5. ABCB7 Transporter

The ATP-binding cassette sub-family B member 7 (ABCB7) is a membrane transport protein located in the mitochondrial inner membrane, on the cytoplasmic membrane, and in the cytoplasm [2,10,17]. ABCB7 has emerged as a critical factor in the pathogenesis of sideroblastic anemia [10]. The ABCB7 gene is located at Xp13.3 and encodes a mitochondrial transporter of the Fe–S cluster [18]. ABCB7 is vital for the maturation of cytosolic and mitochondrial Fe-S cluster proteins besides its involvement in other cellular functions such as DNA repair and nucleotide excision repair, oxidative DNA damage repair, ribosome biogenesis, and tRNA thiol-modification [19]. Iron–sulfur (Fe-S) clusters play a vital role as cofactors for various enzymes, such as DNA polymerases, helicases, and glycosylases [20]. These clusters are initially synthesized as Fe-S intermediates within the mitochondria and subsequently transported to the cytoplasm for maturation by the mitochondrial transporter ABCB7 [18,20].

Though the mechanism of the role of ABCB7 in heme synthesis is still elusive, several studies have proposed its role in heme synthesis and mitochondrial iron retention [18,20,21]. During heme synthesis, ALA, formed from the condensation of glycine and succinyl-CoA, is transported outside the mitochondria and catalyzed to form coproporphyrinogen III. After the subsequent reactions, ferrous iron is incorporated into protoporphyrinogen IX to form heme, a reaction catalyzed by ferrochelatase. The transferrin receptor (TMEM14C) has been suggested to be responsible for transporting protoporphyrinogen IX into the mitochondria [18]. Furthermore, HSPA9 and GLRX5 are among the Fe-S cluster assembly complexes responsible for generating the Fe–S cluster [18]. Finally, ABCB7 exports the Fe-S cluster into the cytosol [2,18,20]. A recent report also demonstrated that ABCB7 is required for bone marrow B cell development, proliferation, and class switch recombination [20]. This study shows that when ABCB7 was conditionally deleted using Mb1-cre, a severe blockage in bone marrow B cell development at the pro-B cell stage was observed [20]. Further investigation indicates that ABCB7 is essential for hematopoiesis [4]. 

ABCB7 mutations cause a rare recessive X-linked sideroblastic anemia with ataxia (XLSA/A) disorder (Figure 1) associated with spinocerebellar ataxia [10,18,21]. The main characteristic of XLSA/A is the early onset of non- or slowly progressive cerebellar ataxia and hypochromic microcytic anemia [20]. ABCB7 is vital for the maturation of cytosolic and mitochondrial Fe-S cluster proteins and the transportation of iron out of the mitochondria [4]. Mutations in the ABCB7 gene have been associated with mitochondrial iron retention, isodicentric X chromosome at Xq12-q13, and sideroblastic anemia [22]. XLSA/A is caused by a few missense mutations identified in ABCB7 [2,23].

The alteration of ABCB7 activity is linked to reduced Fe–S cluster-dependent enzyme activities [18]. Specifically, a recent study suggests that XLSA/A is caused by an impairment of the maturation of cytosolic Fe-S proteins. ABCB7 contributes to the export of the matured Fe/S clusters produced in the mitochondria to be assembled in the cytoplasm. Therefore, a combination of ABCB7 mutations and impaired Fe-S maturation blocks the export of Fe-S and results in mitochondrial iron overload [4,10].

A bone marrow examination of patients with XLSA/A revealed ringed sideroblasts [21]. Several studies have reported a strong relationship between increasing the percentage of bone marrow ringed sideroblasts and decreasing ABCB7 gene expression levels [24,25]. In an in vitro study, knockdown of the ABCB7 gene resulted in a time-dependent loss of mitochondrial Fe-S proteins, ferrochelatase, glutaredoxi 5 (GLRX5), and multiple subunits of the respiratory complexes I and II (NDUFS1 and NDUFS8 in complex I and SDHB in complex II) in all the cell lines tested [21]. The authors further show that loss of ABCB7 altered cellular iron distribution and induced apoptosis of erythroid progenitor cells [21]. These studies corroborate findings reported in human subjects with XLSA/A sideroblastic anemia [10]. The clinical features of ABCB7 mutation-induced sideroblastic anemia are a direct result of the molecular structure and nature of the mutation of the gene.

## 6. Molecular Dynamics of ABCB7 in the Etiology of Sideroblastic Anemia

The molecular structure, architecture, as well as type of mutation in the ABCB7 gene greatly impact the uniqueness of clinical ramifications of XLSA/A compared to XLSA and other variant forms of sideroblastic anemia. Five major clinical manifestations primarily distinguish XLSA/A from XLSA. XLSA/A, which is associated with the long arm of the X-chromosome (Xq13.3, OMIM 301300), manifests as high levels of erythrocyte free protoporphyrin, poor coordination, restricted tendon reflex, normal iron stores, and unresponsiveness to pyridoxine [26]. Conversely, XLSA is linked with the short arm of the X-chromosome (Xp11.21, OMIM 301300) and usually resolves with pyridoxine supplementation. Further, XLSA is often devoid of the neurological defects that are associated with XLSA/A [26].

Using a macrophage cDNA library, the ABCB7 gene was originally isolated by Savary et al. from mice as a 2684 bp cDNA [27]. The ATG codon began from 196 bp and the full novel isolate was purported to encode a protein of 692 amino acids long with 6 transmembrane and other domains as a classical half transporter of ABC [28]. Interestingly, not only did the mouse ABCB7 gene show a 92% sequence identity with a 1109 bp cDNA obtained from an Expressed Sequence Tag database for fetal liver, but its first 346 C-terminal amino acids product shared a 94% similarity with the protein encoded by the fetal liver cDNA [27,28]. Extensive RNA analyses revealed a vast tissue distribution encompassing but not limited to the human placenta, skeletal muscle, pancreas, liver, lung, and heart, with the exception of the brain. These results were mostly corroborated by animal models through deletion studies and X-chromosome inactivation [28]. Furthermore, mouse models showed that the removal of the maternally inherited ABCB7 gene was fatal to growing embryos due to the retention of X-chromosome by extraembryonic tissue, indicating the gene is essential for the development of the fetus. From about the 6th day, the ABCB7 gene is expressed through the different stages of the mouse embryo, according to data from Northern blot studies [28]. However, knocking out the ABCB7 gene in the liver only led to mild mitochondrial damage and impaired iron metabolism [28]. The human ABCB7 gene shows the highest sequence homology with two species of yeast, namely, Schizosaccharomyces pompe (HTM1 gene) and saccharomyces cerevisiae (ATM1 gene) [27,28]. These reports were confirmed by Shimada et al., who found a strong sequence similarity of the human ABCB7 gene with the mouse Abcb7 and yeast ATM1 gene [28,29,30,31]. They concluded that the 2.6 kb human ABCB7 gene is a half transporter that transports heme from the mitochondria to the cytosol [28]. The extensive tissue distribution and sequence homology across different domains of life lay credence to the evolutionary preservation and functional importance of the ABCCB7 gene.

The first mutation in the ABCB7 gene was discovered in 1999 in a family that showed an inheritance pattern of XLSA/A [32]. Other mutations related to XLSA/A have since been identified in ABCB7 and its homologues. It has been abundantly shown that the wild-type ABCB7 gene is primarily involved in the cytosolic assembly of the Fe/S cluster of proteins, whereas the mutant variant loses this functionality. Phenotypically, this specific loss of function, which also impairs the maturation of the cytosol-based Fe/S proteins, is responsible for the unique symptoms experienced by XLSA/A patients [32].

Bekri et al. characterized the molecular defect of ABCB7 in a proband and his family members. The group first determined the molecular structure of the genomic DNA and compared it through gene amplification with the cDNA obtained from the reverse RNA transcript. The study, by using the same primer for DNA amplification as with gene sequencing, revealed that the ABCB7 gene is made up of 16 exons in the cDNA that matched 2356 nucleotides in the genomic DNA (GenBank accession numbers AF241872 to AF241887) [26,28]. Mismatch PCR analysis has been employed to demonstrate a single-gene nucleotide G-C mutation in intron 10 (3rd nucleotide). In addition, Xbal restriction yielded two products (30 bp and 145 bp) in the presence of base C but undigested 175 bp in the presence of base G [26]. However, occurring in an intron of the genomic DNA, this rare nonsense mutation likely has no consequential phenotype or protein product.

Originally mapped between Xq12-q13 using In Situ Hybridization, the localization of the human ABCB7 gene fine-tuned to Xq13.1-q13.3 with a more advanced Fluorescence In Situ Hybridization [28]. Years of molecular genetic studies, molecular cloning, genetic sequencing, genetic mapping, family studies, and mutagenesis culminated in the distinctive identification of three mutations and allelic variants underlying the etiology of XLSA/A. The first identified was the T to G missense mutation at nucleotide position 1400 in the 5th transmembrane domain, Ile400Met (XLSA/A, 0.0001, 301310); the second was a G to A missense mutation at exon 10 nucleotide position 1305, Lys433Glu (XLSA/A, 0.0002, 301310); and the third was a transversion from G to C at cDNA position 1299, Val411Leu (XLSA/A, 0.0003, 301310) [28]. While the mutations are different, each of them led to similar phenotypes and clinical manifestations: anemia, sideroblastic anemia, and cerebellar ataxia, hence qualifying as XLSA/A disease.

Other novel genetic variants of ABCB7 that manifest different phenotypes have been reported in the literature. For instance, in 2016, Protasova et al. published a clinical case of cerebellar hypoplasia and ataxia in which the affected males from the Buryat family had inherited a previously unreported X-linked missense mutation in exon 16 of ABCB7 and, simultaneously, an X-linked 41.4 kb deletion in exon 2 of the ATP7A, a copper transporter [33,34,35]. It was revealed that a C to T substitution in the nucleotide-binding domain of the ABCB7 gene was responsible for the mutation, and this resulted in the wild-type glycine being replaced with serine (Gly682Ser) in the gene product. As a negative control, no mutation or deletion was found at the same gene locus for both ABCB7 and ATP7A in unrelated healthy individuals who did not manifest the clinical symptoms. The healthy subjects were also screened for G to A mutation in the ATP2B3 gene (p.Gly1107Asp), previously associated with cerebellar ataxia, but were found to be free of the mutation [33]. Even though previous genetic variants exhibiting sideroblastic anemia with cerebellar ataxia-related ABCB7 mutations (XLSA/A) were localized in the transmembrane domain, this mutation was found in the nucleotide-binding domain inside the inner space of the mitochondrion, suggesting that this mutation could hinder the proteins’ ability to bind to other cofactors. Notably, disruption of the gene at the same locus in the yeast ABCB7 gene orthologue, ATM1, resulted in a loss of functional activity and iron accumulation [31,33]. The evaluated patients manifested some of the symptoms of classic XLSA/A, cerebellar and nonprogressive ataxia, and neurological and mental disorders. Remarkably, though, the patients did not manifest clinical features of sideroblastic anemia, which the authors attributed to either the peculiarity of this kind of ABCB7 mutation or the consequences of its combination with the ATP7A gene’s deletion [33]. Patients with only this type of genetic deletion typically experience severe pediatric neurological disease and even early childhood mortality. Interestingly, in the presence of the ABCB7 mutation, the affected males did not show such serious illness. The exact role of the ABCB7 gene in mitigating such clinical manifestations or that of the ATP7A gene in ridding patients of sideroblastic anemia is unclear. Based on the most recent literature, no other novel XLSA/A-like early onset cerebellar ataxia-causing mutant variants have been found in ABCB7 but have been found in other genes, such as the LRCH2 gene and CSMD1 [34].

Reports from knockout cell line studies of the bridging between ABCB7 and ABCB10 connected by ferrochelatase suggest a potential partnership between both transporters in the development of XLSA/A sideroblastic anemia [21]. One study, which characterized all three proteins, found that ABCB7 and ABCB10 are homodimers requiring pairing through connection by the dimeric ferrochelatase in proximity with each of their nucleotide binding domains [21]. The authors found that the importation of iron by the mitochondria depended on the interaction of ABCB10 and ferochelatase [21], and this may open opportunities for understanding the complex mechanism of the architectural and functional concert between ABCB7 and ABCB10 in the development of XLSA/A sideroblastic anemia. The role of ABCB7 in sideroblastic anemia highlights its importance in mitochondrial iron transport and heme synthesis.

## 7. Clinical Consequences of Sideroblastic Anemia

The mitochondria of patients with sideroblastic anemia are characterized by the accumulation of iron around the nucleus of the mitochondrial erythroblasts in bone marrow aspirate smear [8]. The clinical presentation and diagnosis of sideroblastic anemia depends on the type, congenital or acquired sideroblastic anemia. Also, congenital sideroblastic anemia can be early or late-onset sideroblastic anemia [8,36]. Diseases caused by congenital sideroblastic anemia can be categorized into nonsyndromic and syndromic. Nonsyndromic conditions include X-linked sideroblastic anemia, also known as sideroblastic anemia 1 (SIDBA1), SIDBA2, SIDBA3, and SIDBA4. Conversely, syndromic diseases include X-linked sideroblastic anemia with ataxia (XLSA/A) caused by ABCB7 mutations; Pearson’s marrow-pancreas syndrome (PMPS), thiamine-responsive megaloblastic anemia (TRMA); myopathy, lactic acidosis, and sideroblastic anemia (MLAS1, MLAS2); sideroblastic anemia with immunodeficiency, fevers, and developmental delay (SIFD); and NDUFB11 deficiency [36].

Generally, the clinical consequences of iron accumulation in the mitochondria include anemia, the formation of ringed sideroblasts, iron overload, fatigue and weakness, pallor, and an enlarged liver or spleen. Individuals with sideroblastic anemia can present with dizziness, heart palpitations, decreased exercise tolerance, and headache, depending on the severity of the anemia induced by iron accumulation. Other clinical presentations might be specific to the type of sideroblastic anemia. Specifically, clinical presentations of XLSA/A caused by ABCB7 mutations present as a childhood onset of non-progressive cerebellar ataxia and mild anemia with hypochromia and microcytosis [23]. Such is the clinical relevance of mutations in ABCB7 that they are found to be involved in several other abnormalities and clinical features. Abnormalities aside from blood abnormalities or hematological abnormalities include sideroblastic anemia; abnormalities of the eye, genitourinary system, integument, musculoskeletal system, nervous system, and voice; growth abnormalities; and autism spectrum disorder [37]. The age of patients at which symptoms have been reported in most cases is less than two years [10]. Patients with XLSA/A interestingly present with mild anemia, not requiring transfusion. However, other presentations include non-progressive ataxia, a delay in motor development, elevated free erythrocyte protoporphyrin levels, and a lack of excessive iron deposition [2,10]. Though the neurological features were non-progressive at the early onset, by the fifth decade, slow progression of the neurological problem is evident [38]. In some cases, ataxia may create difficulties in sitting, standing, and walking for the patient without assistance. Other individuals may experience an impaired ability to judge distances (dysmetria) and struggle with rapid movements (dysdiadochokinesis) and poorly coordinated movements. Other impacted patients have been reported to endure irregular eye movements, moderate speech struggle, or dysarthria and tremors [37]. Interestingly, Xiong et al. reported epilepsy and cryptorchidism, unique clinical presentations uncommon in patients with XLSA/A anemia at early onset [10]. This suggests that the clinical manifestations of sideroblastic anemia vary based on their severity and gene mutations.

## 8. Diagnosis of Sideroblastic Anemia

Identifying the underlying cause of sideroblastic anemia is vital to differentiating it from other forms of anemia. Some of the workups to diagnose sideroblastic anemia include the complete blood count (CBC), bone marrow aspiration and biopsy, peripheral smear, iron studies, and genetic testing and genotyping [3]. Prussian blue or Perl’s reaction is used to stain blood or bone marrow specimens for peripheral blood smears [36]. The presence of ringed sideroblasts in the bone marrow smears confirms the diagnosis of sideroblastic anemia. Patients (especially those with mutations in XLSA, GLRX5, and SLC25A38) might present with signs of anemia, systemic iron overload, and elevated iron concentrations in the liver that may even render the liver cirrhotic [36]. Magnetic Resonance Imaging may be utilized to noninvasively evaluate cardiac and hepatic iron overload [39]. This patient group may also have high ferritin, usually higher than those with ALA2 mutation [40]. The hereditary forms of sideroblastic anemia usually present with microcytic anemia, while acquired forms often present with macrocytic anemia [8] or normocytic anemia in some cases. Also, due to iron overload, patients with sideroblastic anemia present with an elevated serum iron concentration, decreased TIBC, normal transferrin, elevated ferritin, and high percent saturation of the iron-binding protein [39,41].

The bone marrow of patients with sideroblastic anemia exhibits significant iron accumulation, increased erythroid cell proliferation with impaired hemoglobin production, and elevated counts of sideroblasts [41]. Genetic testing, such as next-generation sequencing, is necessary for congenital sideroblastic anemia and acquired forms after the reversible causes have been ruled out [3]. However, combining genetic testing with a bone marrow smear is crucial to confirm a diagnosis [42]. XSLA/A X-linked inheritance with ataxia, associated with ABCB7 is so rare only six cases have been described in the literature. In the clinical case of a 5-year-old male patient with a history of anemia, XSLA/A ataxia, and of Chinese descent, extensive laboratory tests, including genetic testing and hereditary investigation, were performed as evidence of diagnosis and to confirm the inheritance pattern of ABCB7-mutation-induced sideroblastic anemia [10]. In general, genetic, clinical testing of consequence in the investigative diagnosis of ABCB7-mutation-induced abnormalities include targeted variant analysis, sequence analysis of the entire coding region, sequence analysis of select exons, and deletion or duplication analysis [37], and next-generation sequencing has played a key role in advancing molecular testing [43].

## 9. Treatment of Sideroblastic Anemia

The treatment of sideroblastic anemia depends on the type and severity of the disease. Also, treating the underlying condition is vital if it is due to a secondary cause [4]. For instance, myelodysplastic syndromes with ring sideroblasts, which are known as the most common acquired sideroblastic anemia, can be treated using red blood cells and platelet transfusions; erythropoiesis-stimulating agents (ESA); immunomodulatory agents, such as lenalidomide; TGF-superfamily members’ regulators; allogeneic stem-cell transplantation; and iron-chelation therapy [4].

However, oral pyridoxine (vitamin B6) at 50–100 mg/day has been proven to partially or completely correct anemia for patients with X-linked sideroblastic anemia [36]. Pyridoxine supplementation has been reported to be effective in genetic mutation-related sideroblastic anemia because of its role as a cofactor in heme synthesis [4,10,41]. Blood transfusion might be indicated in severe cases for patients who are not responsive to pyridoxine [36]. It is essential to monitor patients receiving blood transfusions for iron overload. If the serum ferritin level exceeds 1000 ng/L, initiating deferoxamine or oral chelator treatment is recommended [36].

## 10. Pharmacogenomics of the Management of ABCB7 Mutation-Induced XLSA/A

As noted, sideroblastic anemia can either be acquired or inherited. While both sources of the disease have nearly similar approaches to treatment, as outlined in the Treatment section of this article, the management of ABCB7 mutation-induced XLSA/A deviates slightly but significantly from the others. This deviation is not merely due to its genetic origin because other mutation-driven sideroblastic anemia diseases are not quite as anomalous. Rather than the kind of gene mutation, it is the specific affected protein (ABCB7) that plays a crucial role in the pharmacogenetics of XLSA/A. Two principal distinguishing features of the therapeutic management of XLSA/A in relation to the other forms of sideroblastic anemia are important to note: the administration of pyridoxine (vitamin B6) for sideroblastic anemia and the supplementation of iron for iron-deficiency anemia. Pyridoxine supplementation has been shown to be effective in alleviating the symptoms of many forms of sideroblastic anemia, such as those caused by pyridoxine deficiency and XLSA [3]. For obvious reasons, the administration of pyridoxine reverses the anemia and eliminates ring sideroblasts in pyridoxine deficiency-induced sideroblastic anemia. Treatment with vitamin B6 also resolves symptoms of sideroblastic anemia in some genetic cases of the disease, such as XLSA. Pyridoxine in the form of pyridoxal phosphate is a known cofactor of ALAS2, the enzyme responsible for the conversion during the first step in heme biosynthesis [44]. Restriction of this step mitigates the formation of 5-aminolevulinate, a required upstream precursor for the eventual incorporation of iron into heme along the pathway of heme synthesis. In XLSA, the mutation in ALA2, which usually occurs either in the pyridoxal phosphate-binding or the catalytic domains [3], causes diminished activity of the enzyme to interact with pyridoxal phosphate. Pyridoxine supplementation can improve the activity of the enzyme and restore iron homeostasis and anemia [45,46]. Interestingly, though XLSA and XLSA/A are both x-linked by inheritance, they display divergent pharmacogenomics.

XLSA/A is unresponsive to pyridoxine administration. In XLSA/A, the cause of the classic sideroblastic anemia symptoms with cerebellar ataxia is due to variable missense mutations in the ABCB7 gene, rather than in the ALA2 gene. These mutations largely impact iron utilization and transport in the mitochondria with inconsequential effects on porphyrin metabolism except for an increase in protoporphyrin [10]. Since pyridoxine is not a requisite cofactor for the protein products encoded by ABCB7, XLSA/A patients typically demonstrate pyridoxine refractory. Some XLSA/A patients with ataxia who reported taking oral vitamin B6 supplements showed no improvement in hematological parameters such as hematocrit, hemoglobin, mean cell volume, reticulocyte count, and mean cell hemoglobin concentration [10]. Due to the pharmacogenomics of ABCB7 mutations in relation to pyridoxine, vitamin B6 is not recommended for the treatment of XLSA/A. Persistent iron overload has been reported to be a causative parameter for refractory pyridoxine, and a reduction in iron via phlebotomy improves the responsiveness to pyridoxine and alleviates anemia in general [3]. Mitochondrial iron overload, which is a participant in the pathogenesis of ABCB7-mediated sideroblastic anemia, may partly be responsible for the unresponsiveness to pyridoxine. A mutation in an unrelated mitochondrial gene (SLC25A38), whose protein is responsible for solute transport, causes sideroblastic anemia and is also refractory to pyridoxine [47]. A rare case of acquired genetic deletion, isodicentric (idicXq13), involves partial or entire loss of the ABCB7 gene since the portion of the X-chromosome localized by the ABCB7 gene is removed. A female patient with the idicXq13 deletion persistently showed unresolved anemia after treatment with pyridoxine [48]. These may indicate a prominent pharmacogenetic relationship between a disruption in the ABCB7 gene and unresponsiveness to pyridoxine. Due to these obstacles discussed, the therapeutic management of XLSA/A and other pyridoxine-refractory forms incorporate supportive care with intermittent red cell transfusions, with a significant percentage of patients being life-long transfusion-dependent [3]. 

Refractory anemia with ring sideroblasts (previously known as RARS) is frequently caused by bone marrow-originated myelodysplastic syndromes (newly designated MDS-RS), and characterized by 15% ring sideroblasts in the bone marrow. Extensive gene-expression studies, DNA sequencing, and methylation experiments have reportedly shown a correlation between low expression of the ABCB7 gene and RARS [4,49]. Individuals with lowly expressed but intact wild-type ABCB7 gene showed refractory anemia [49]. This suggests that the ABCB7 gene may play a role in the development and exacerbation of RARS in the presence or absence of mutation. Furthermore, ABCB7 is highly expressed in the bone marrow [10], the same tissue where MDS typically originates. While the principal cause of refractory deficiency anemia with ring sideroblasts is the bone marrow MDS involvement in the pathogenesis of sideroblastic anemia, understanding the role of ABCB7 is imperative. Since ABCB7-related XLSA/A is a genetic form and MDS-related XLSA/A is a primary acquired form of the disease, this phenomenon may be one of many interplays between the environmental and genetic causes of sideroblastic anemia.

A third refractory disorder could be postulated to have a potential ABCB7 correlation. Iron-refractory iron deficiency anemia is a rare genetic (*TMPRSS6* gene encoding Matriptase-2) condition in which iron deficiency anemia is unresponsive to the oral supplementation of iron but can also be acquired [50,51]. This is particularly important because even though sideroblastic anemia, in general, is not discriminatory among microcytic, normocytic, and macrocytic anemia, ABCB7-related sideroblastic anemia with ataxia (XLSA/A) is often associated with microcytic anemia [2,52]. XLSA/A-mediated poor iron utilization, against the background of excessive mitochondrial iron storage, could be the cause of this observation and may also render oral iron supplementation ineffective. As noted in Figure 2, genetic testing is indicated in the setting of ring sideroblasts and microcytic anemia refractory to iron. This arguably suggests that iron therapy may not improve microcytic anemia in the setting of XLSA/A. The literature is scant and inconclusive on this interesting pharmacogenomic linkage. Further studies are recommended to ascertain the nature of the relationship between iron-refractory iron deficiency anemia and XLSA/A-caused mutations in the ABCB7 gene.

## 11. Conclusions

Sideroblastic anemia is a rare hematological condition that can have severe complications, including pancytopenia and ataxia, that may require lifelong management with medications, bone marrow biopsy-based tests, and other procedures. ABCB7, a transmembrane Fe-S cluster mitochondrial protein belonging to the family of ATP-dependent transporters, plays a role in the etiology and pathogenesis of the rarest forms of sideroblastic anemia. This review article is an essential scholarly effort to shed light on both the disease and the responsible mutations responsible, in addition to exogenous causes. The current article is expected to generate scientific, medical, and research efforts toward appropriate diagnostic testing, including molecular technologies, and management of the disease. As protein researchers advance the molecular characteristics of ABCB7, its association with sideroblastic anemia will further be unraveled, as well as unwind the possibility of other genetic factors that may play key roles in the pathophysiology of the disease.

## Figures and Tables

**Table 1:** Types, causes, pathogenesis and diagnostic workup of sideroblastic anemia.

Type	Cause	Pathogenesis	Diagnostic Testing
Congenital sideroblastic anemia			
X-linked mutations	*ABCB7*	Assembly/transport of Fe-S clusters containing hemoglobin stalls	CBC, PBS, Fe studies, Genetic testing
	*ALAS2*	Being the 1st enzyme in heme synthesis, the process disrupts	
	*NDUFB11*	Impaired NADPH subunit disrupts oxidative phosphorylation	
Maternal inheritance	Mitochondrial DNA *MT-ATP6*	Protein synthesis is hampered. ATPase-dependent oxidative phosphorylation is affected	CBC, PBS, Fe studies, Genetic testing
Autosomal recessive mutations	*SLC19A2*, *SLC25A38*, *TRNT1*, *GLRX5*, *HSPA9*, *HSCB*, *FECH*, *PUS1*, *LARS2*, *YARS2*	Impairment of Fe-S cluster formation (HSCB) or incorporation of Fe into heme (FECH) or mitochondrial protein synthesis (YARS2)	CBC, PBS, Fe studies, Genetic testing
Acquired sideroblastic anemia			
MDS/MPN	*SF3B1* mutation *JAK2V617F* mutation	*SF3B1* impairs mRNA splicing involved with heme synthesis	PBS, Bone marrow biopsy, F.I.S.H.
Hypothermia		Disrupts oxidative phosphorylation	
Heavy metals	Copper deficiency	Mitochondrial iron builds up when Cu, a superoxide dismutase cofactor, is low	Fe studies heavy metal testing (Cu, Pb, Zn)
	Lead poisoning	Pb inhibits FECH required for incorporating Fe into heme	
	Zinc toxicity	Zn, rather than Fe, is incorporated in heme, and Fe accumulates in mitochondria	
Medications	Isoniazid	Interferes with ALAS2 function by inhibiting pyridoxine metabolism	Patient medication history, Drug testing, TDM
	Linezolid	Inhibits mitochondrial protein synthesis	
	Triethylenetetramine hydrochloride	Used to treat Wilson’s disease (genetic disease that causes copper overload). Overtreatment can cause copper deficiency	
	Chloramphenicol	Diminishes cytochrome activity by impeding mitochondrial respiratory function	
Alcohol		Disrupts pyridoxine metabolism	Patient history, alcohol testing
Vitamin B6	B6 deficiency	Heme synthesis is dependent on pyridoxal phosphate as enzyme cofactor for ALAS2	B6 testing

## Data Availability

Not applicable.
